# Supplementary material for: Genome-wide meta-analysis identifies eight new susceptibility loci for cutaneous squamous cell carcinoma
Source: Nat Commun. 2020 Feb 10;11:820. doi: 10.1038/s41467-020-14594-5 (PMC7010741; doi:10.1038/s41467-020-14594-5)
Supplement: Supplementary file 2 — Supplementary Information [file 41467_2020_14594_MOESM2_ESM.pdf]

Genome-wide meta-analysis identifies eight new susceptibility loci for cutaneous squamous cell carcinoma

Sarin et al.

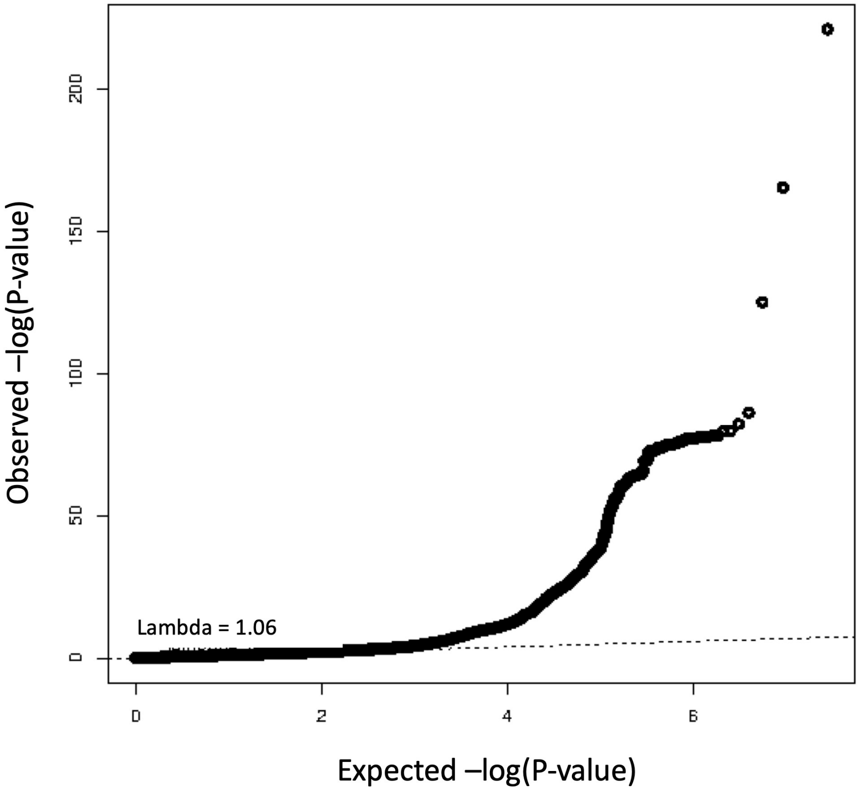

Supplementary Figure 1: QQ plot for SCC Meta-analysis

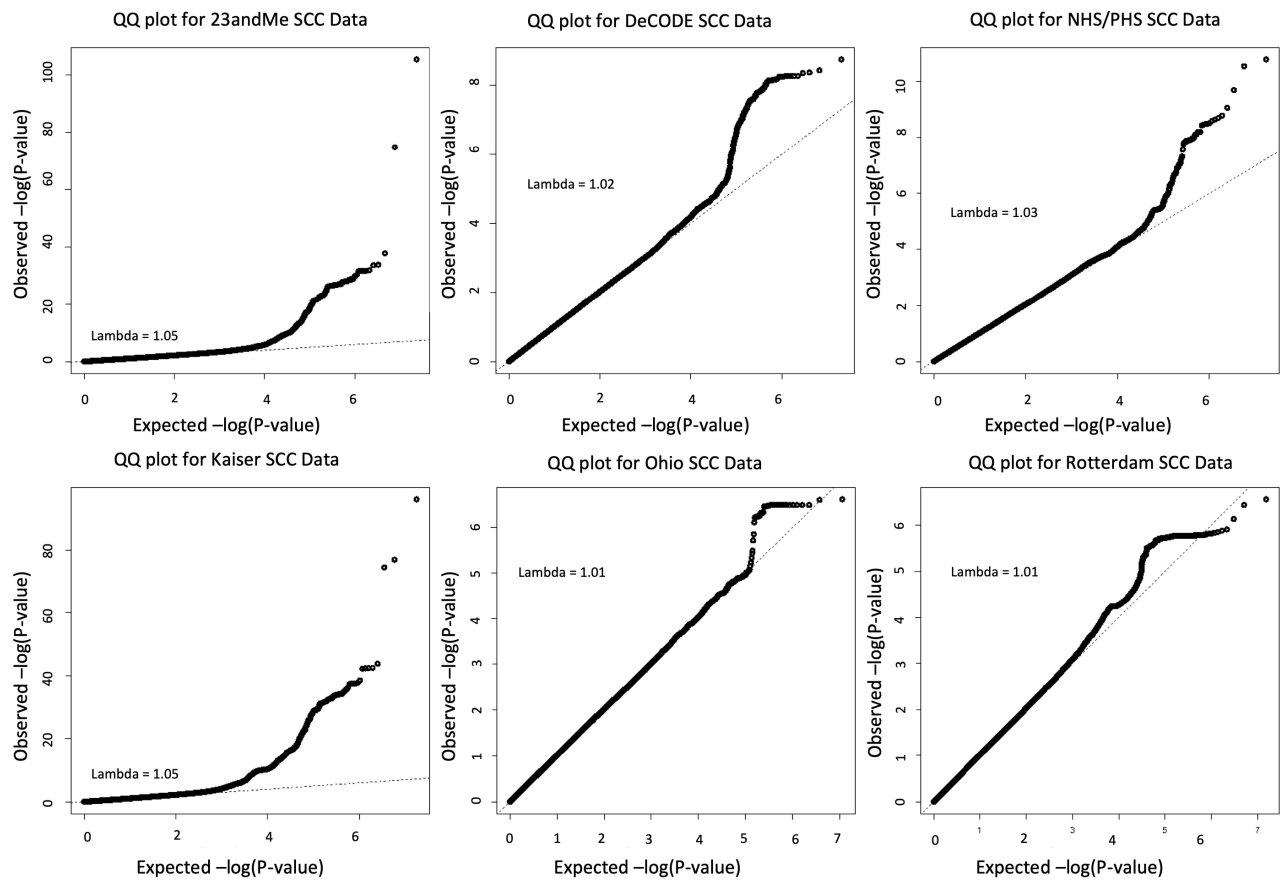

Supplementary Figure 2: QQ plot by individual GWAS study used in meta-analysis

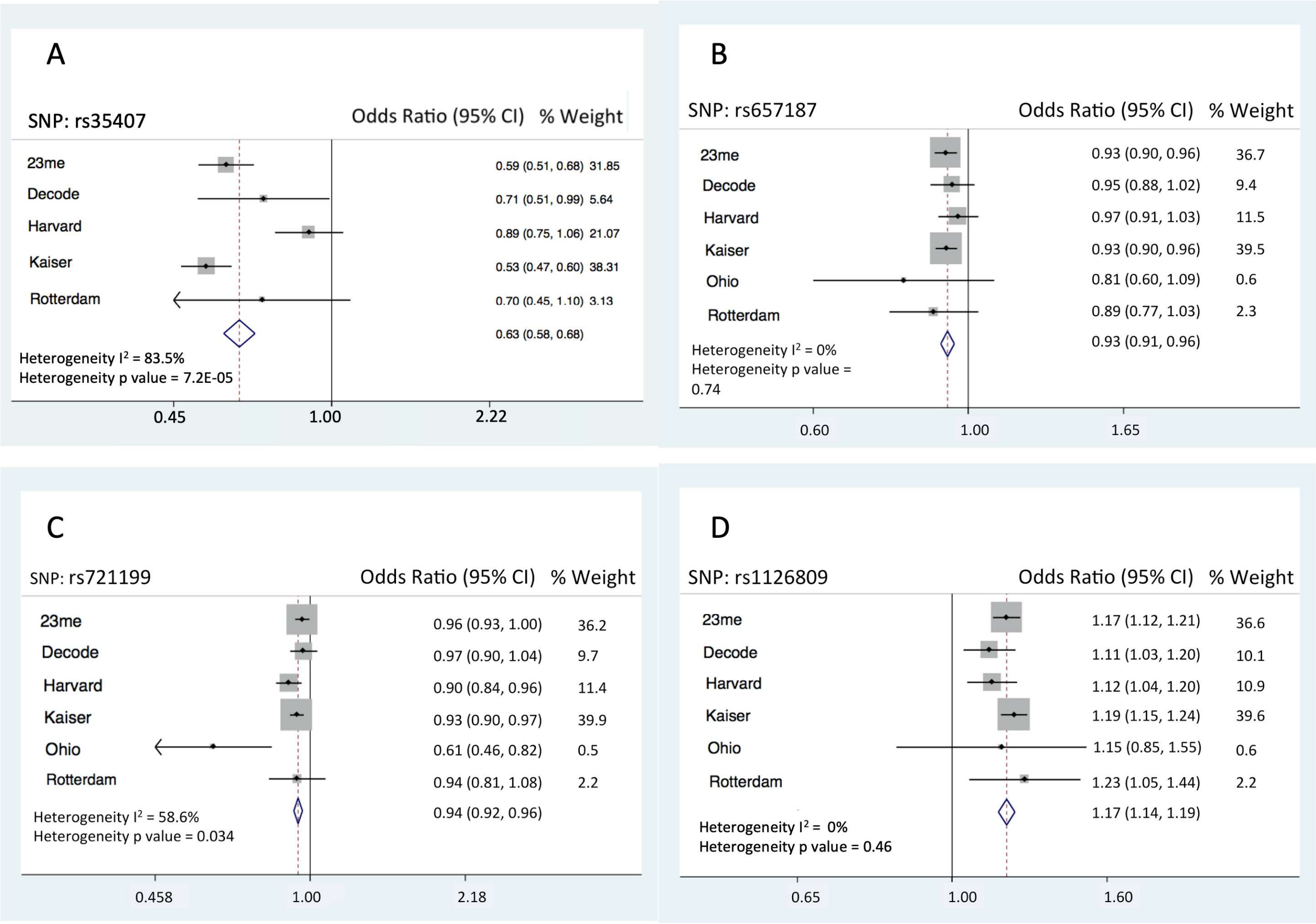
A
SNP: rs35407
Odds Ratio (95% CI) % Weight
23me 0.59 (0.51, 0.68) 31.85
Decode 0.71 (0.51, 0.99) 5.64
Harvard 0.89 (0.75, 1.06) 21.07
Kaiser 0.53 (0.47, 0.60) 38.31
Rotterdam 0.70 (0.45, 1.10) 3.13
0.63 (0.58, 0.68)
Heterogeneity I2 = 83.5%
Heterogeneity p value = 7.2E-05
0.45
1.00
2.22
B
SNP: rs657187
Odds Ratio (95% CI) % Weight
23me 0.93 (0.90, 0.96) 36.7
Decode 0.95 (0.88, 1.02) 9.4
Harvard 0.97 (0.91, 1.03) 11.5
Kaiser 0.93 (0.90, 0.96) 39.5
Ohio 0.81 (0.60, 1.09) 0.6
Rotterdam 0.89 (0.77, 1.03) 2.3
0.93 (0.91, 0.96)
Heterogeneity I2 = 0%
Heterogeneity p value = 0.74
0.60
1.00
1.65
C
SNP: rs721199
Odds Ratio (95% CI) % Weight
23me 0.96 (0.93, 1.00) 36.2
Decode 0.97 (0.90, 1.04) 9.7
Harvard 0.90 (0.84, 0.96) 11.4
Kaiser 0.93 (0.90, 0.97) 39.9
Ohio 0.61 (0.46, 0.82) 0.5
Rotterdam 0.94 (0.81, 1.08) 2.2
0.94 (0.92, 0.96)
Heterogeneity I2 = 58.6%
Heterogeneity p value = 0.034
0.458
1.00
2.18
D
SNP: rs1126809
Odds Ratio (95% CI) % Weight
23me 1.17 (1.12, 1.21) 36.6
Decode 1.11 (1.03, 1.20) 10.1
Harvard 1.12 (1.04, 1.20) 10.9
Kaiser 1.19 (1.15, 1.24) 39.6
Ohio 1.15 (0.85, 1.55) 0.6
Rotterdam 1.23 (1.05, 1.44) 2.2
1.17 (1.14, 1.19)
Heterogeneity I2 = 0%
Heterogeneity p value = 0.46
0.65
1.00
1.60

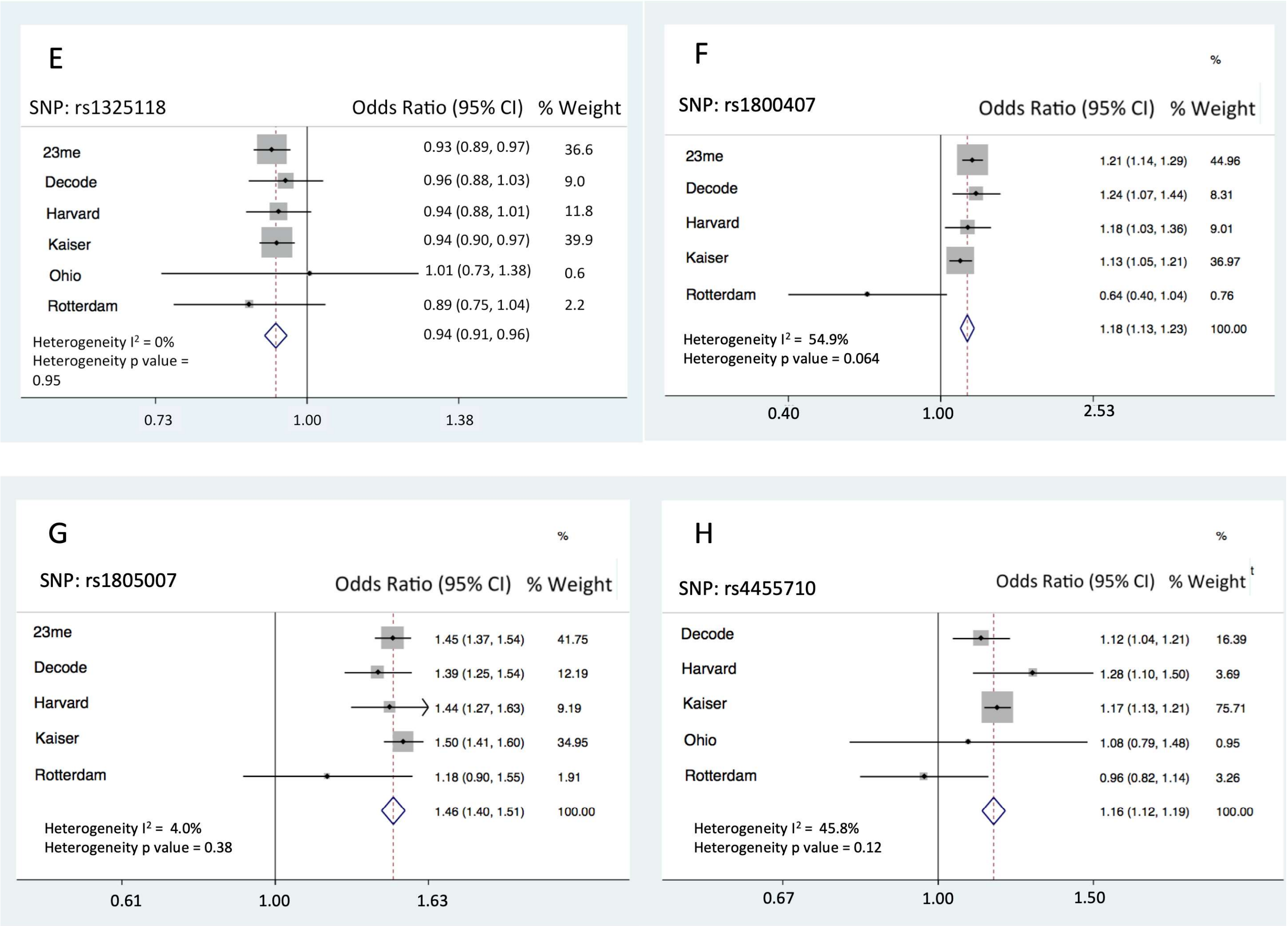
E
SNP: rs1325118
Odds Ratio (95% CI)
% Weight
23me
0.93 (0.89, 0.97)
36.6
Decode
0.96 (0.88, 1.03)
9.0
Harvard
0.94 (0.88, 1.01)
11.8
Kaiser
0.94 (0.90, 0.97)
39.9
Ohio
1.01 (0.73, 1.38)
0.6
Rotterdam
0.89 (0.75, 1.04)
2.2
Heterogeneity I² = 0%
Heterogeneity p value = 0.95
0.94 (0.91, 0.96)
0.73
1.00
1.38
F
SNP: rs1800407
Odds Ratio (95% CI)
% Weight
23me
1.21 (1.14, 1.29)
44.96
Decode
1.24 (1.07, 1.44)
8.31
Harvard
1.18 (1.03, 1.36)
9.01
Kaiser
1.13 (1.05, 1.21)
36.97
Rotterdam
0.64 (0.40, 1.04)
0.76
Heterogeneity I² = 54.9%
Heterogeneity p value = 0.064
1.18 (1.13, 1.23)
100.00
0.40
1.00
2.53
G
SNP: rs1805007
Odds Ratio (95% CI)
% Weight
23me
1.45 (1.37, 1.54)
41.75
Decode
1.39 (1.25, 1.54)
12.19
Harvard
1.44 (1.27, 1.63)
9.19
Kaiser
1.50 (1.41, 1.60)
34.95
Rotterdam
1.18 (0.90, 1.55)
1.91
1.46 (1.40, 1.51)
100.00
Heterogeneity I² = 4.0%
Heterogeneity p value = 0.38
0.61
1.00
1.63
H
SNP: rs4455710
Odds Ratio (95% CI)
% Weight
Decode
1.12 (1.04, 1.21)
16.39
Harvard
1.28 (1.10, 1.50)
3.69
Kaiser
1.17 (1.13, 1.21)
75.71
Ohio
1.08 (0.79, 1.48)
0.95
Rotterdam
0.96 (0.82, 1.14)
3.26
1.16 (1.12, 1.19)
100.00
Heterogeneity I² = 45.8%
Heterogeneity p value = 0.12
0.67
1.00
1.50

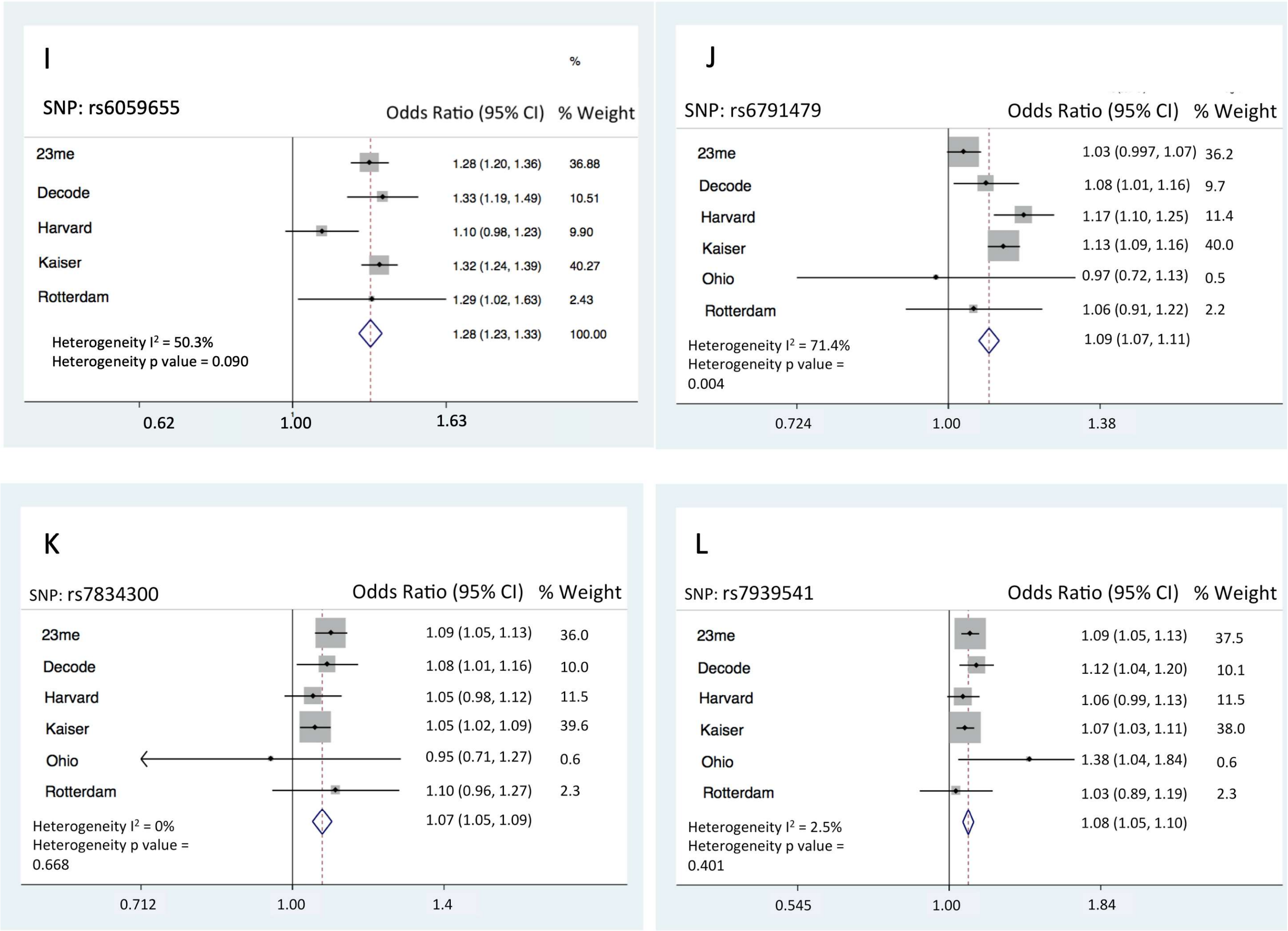
I
SNP: rs6059655
Odds Ratio (95% CI)
% Weight
23me
1.28 (1.20, 1.36)
36.88
Decode
1.33 (1.19, 1.49)
10.51
Harvard
1.10 (0.98, 1.23)
9.90
Kaiser
1.32 (1.24, 1.39)
40.27
Rotterdam
1.29 (1.02, 1.63)
2.43
Heterogeneity I2 = 50.3%
Heterogeneity p value = 0.090
1.28 (1.23, 1.33)
100.00
0.62
1.00
1.63
J
SNP: rs6791479
Odds Ratio (95% CI)
% Weight
23me
1.03 (0.997, 1.07)
36.2
Decode
1.08 (1.01, 1.16)
9.7
Harvard
1.17 (1.10, 1.25)
11.4
Kaiser
1.13 (1.09, 1.16)
40.0
Ohio
0.97 (0.72, 1.13)
0.5
Rotterdam
1.06 (0.91, 1.22)
2.2
Heterogeneity I2 = 71.4%
Heterogeneity p value = 0.004
1.09 (1.07, 1.11)
0.724
1.00
1.38
K
SNP: rs7834300
Odds Ratio (95% CI)
% Weight
23me
1.09 (1.05, 1.13)
36.0
Decode
1.08 (1.01, 1.16)
10.0
Harvard
1.05 (0.98, 1.12)
11.5
Kaiser
1.05 (1.02, 1.09)
39.6
Ohio
0.95 (0.71, 1.27)
0.6
Rotterdam
1.10 (0.96, 1.27)
2.3
Heterogeneity I2 = 0%
Heterogeneity p value = 0.668
1.07 (1.05, 1.09)
0.712
1.00
1.4
L
SNP: rs7939541
Odds Ratio (95% CI)
% Weight
23me
1.09 (1.05, 1.13)
37.5
Decode
1.12 (1.04, 1.20)
10.1
Harvard
1.06 (0.99, 1.13)
11.5
Kaiser
1.07 (1.03, 1.11)
38.0
Ohio
1.38 (1.04, 1.84)
0.6
Rotterdam
1.03 (0.89, 1.19)
2.3
Heterogeneity I2 = 2.5%
Heterogeneity p value = 0.401
1.08 (1.05, 1.10)
0.545
1.00
1.84

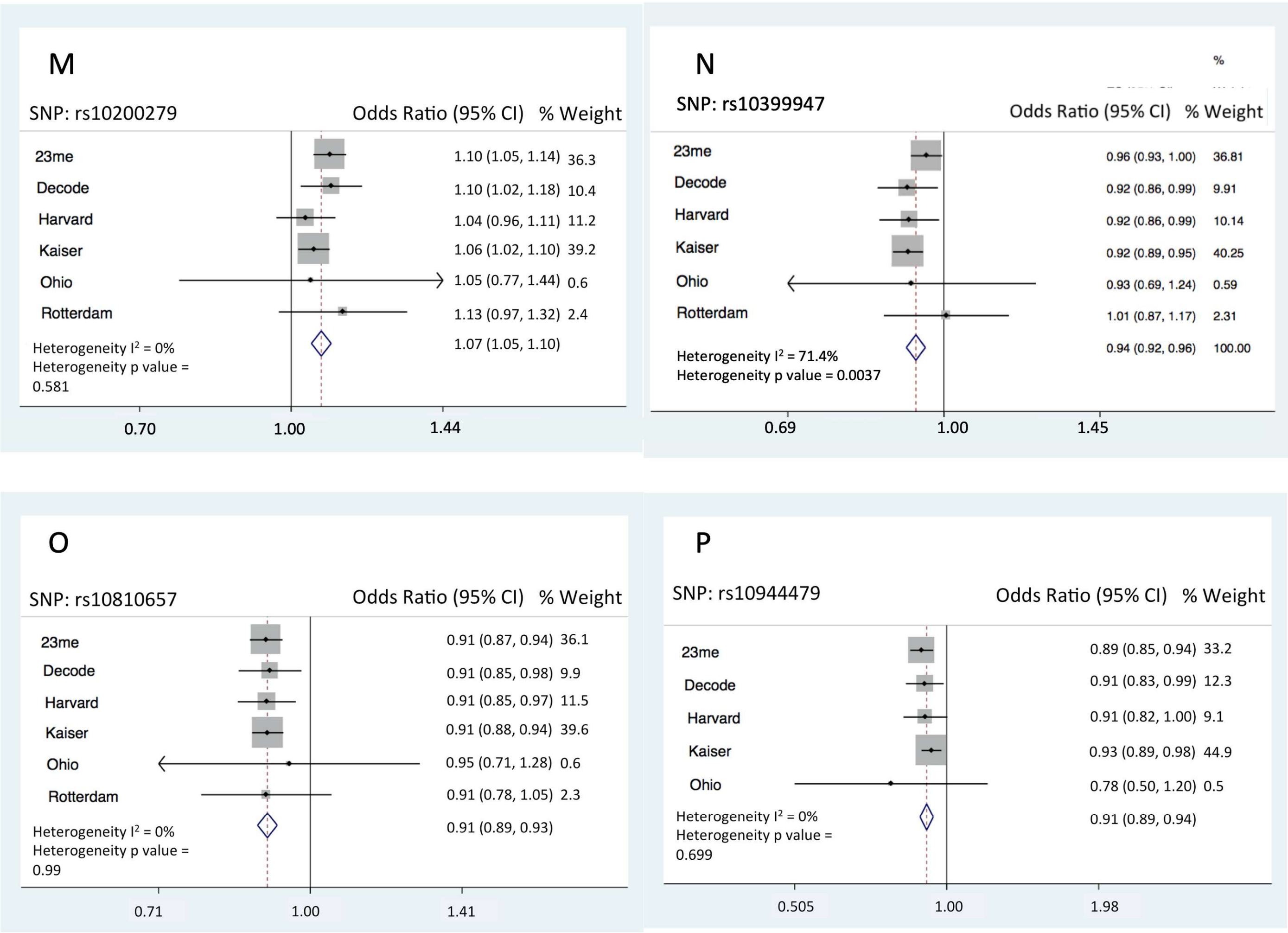
M
SNP: rs10200279
Odds Ratio (95% CI) % Weight
23me 1.10 (1.05, 1.14) 36.3
Decode 1.10 (1.02, 1.18) 10.4
Harvard 1.04 (0.96, 1.11) 11.2
Kaiser 1.06 (1.02, 1.10) 39.2
Ohio 1.05 (0.77, 1.44) 0.6
Rotterdam 1.13 (0.97, 1.32) 2.4
Heterogeneity I² = 0% 1.07 (1.05, 1.10)
Heterogeneity p value = 0.581
0.70 1.00 1.44
N
%
SNP: rs10399947
Odds Ratio (95% CI) % Weight
23me 0.96 (0.93, 1.00) 36.81
Decode 0.92 (0.86, 0.99) 9.91
Harvard 0.92 (0.86, 0.99) 10.14
Kaiser 0.92 (0.89, 0.95) 40.25
Ohio 0.93 (0.69, 1.24) 0.59
Rotterdam 1.01 (0.87, 1.17) 2.31
0.94 (0.92, 0.96) 100.00
Heterogeneity I² = 71.4%
Heterogeneity p value = 0.0037
0.69 1.00 1.45
O
SNP: rs10810657
Odds Ratio (95% CI) % Weight
23me 0.91 (0.87, 0.94) 36.1
Decode 0.91 (0.85, 0.98) 9.9
Harvard 0.91 (0.85, 0.97) 11.5
Kaiser 0.91 (0.88, 0.94) 39.6
Ohio 0.95 (0.71, 1.28) 0.6
Rotterdam 0.91 (0.78, 1.05) 2.3
Heterogeneity I² = 0% 0.91 (0.89, 0.93)
Heterogeneity p value = 0.99
0.71 1.00 1.41
P
SNP: rs10944479
Odds Ratio (95% CI) % Weight
23me 0.89 (0.85, 0.94) 33.2
Decode 0.91 (0.83, 0.99) 12.3
Harvard 0.91 (0.82, 1.00) 9.1
Kaiser 0.93 (0.89, 0.98) 44.9
Ohio 0.78 (0.50, 1.20) 0.5
Heterogeneity I² = 0% 0.91 (0.89, 0.94)
Heterogeneity p value = 0.699
0.505 1.00 1.98

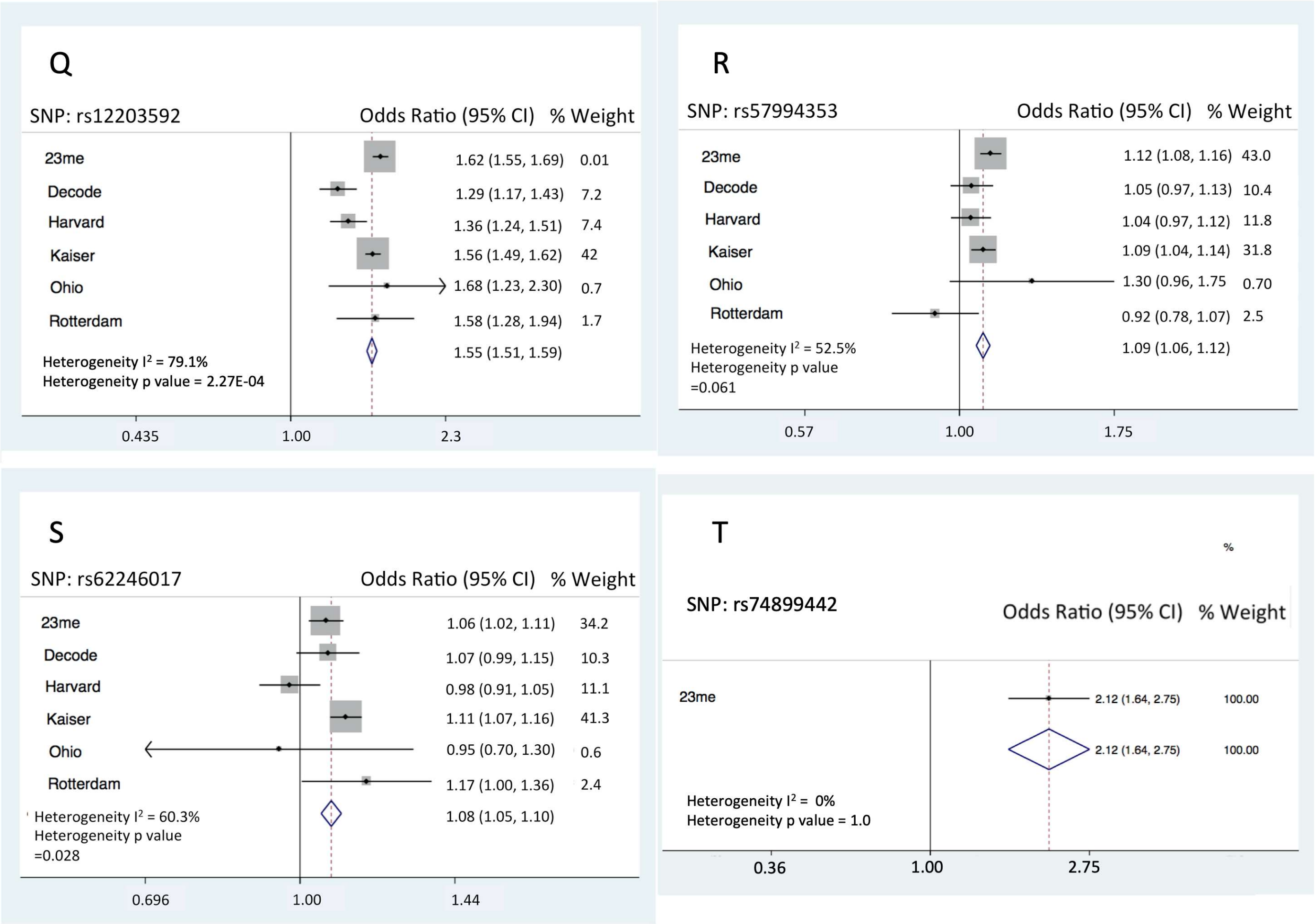

Q
SNP: rs12203592
Odds Ratio (95% CI)
% Weight
23me
1.62 (1.55, 1.69)
0.01
Decode
1.29 (1.17, 1.43)
7.2
Harvard
1.36 (1.24, 1.51)
7.4
Kaiser
1.56 (1.49, 1.62)
42
Ohio
1.68 (1.23, 2.30)
0.7
Rotterdam
1.58 (1.28, 1.94)
1.7
1.55 (1.51, 1.59)
Heterogeneity I2 = 79.1%
Heterogeneity p value = 2.27E-04
0.435
1.00
2.3
R
SNP: rs57994353
Odds Ratio (95% CI)
% Weight
23me
1.12 (1.08, 1.16)
43.0
Decode
1.05 (0.97, 1.13)
10.4
Harvard
1.04 (0.97, 1.12)
11.8
Kaiser
1.09 (1.04, 1.14)
31.8
Ohio
1.30 (0.96, 1.75)
0.70
Rotterdam
0.92 (0.78, 1.07)
2.5
Heterogeneity I2 = 52.5%
Heterogeneity p value
=0.061
1.09 (1.06, 1.12)
0.57
1.00
1.75
S
SNP: rs62246017
Odds Ratio (95% CI)
% Weight
23me
1.06 (1.02, 1.11)
34.2
Decode
1.07 (0.99, 1.15)
10.3
Harvard
0.98 (0.91, 1.05)
11.1
Kaiser
1.11 (1.07, 1.16)
41.3
Ohio
0.95 (0.70, 1.30)
0.6
Rotterdam
1.17 (1.00, 1.36)
2.4
Heterogeneity I2 = 60.3%
Heterogeneity p value
=0.028
1.08 (1.05, 1.10)
0.696
1.00
1.44
T
%
SNP: rs74899442
Odds Ratio (95% CI)
% Weight
23me
2.12 (1.64, 2.75)
100.00
2.12 (1.64, 2.75)
100.00
Heterogeneity I2 = 0%
Heterogeneity p value = 1.0
0.36
1.00
2.75

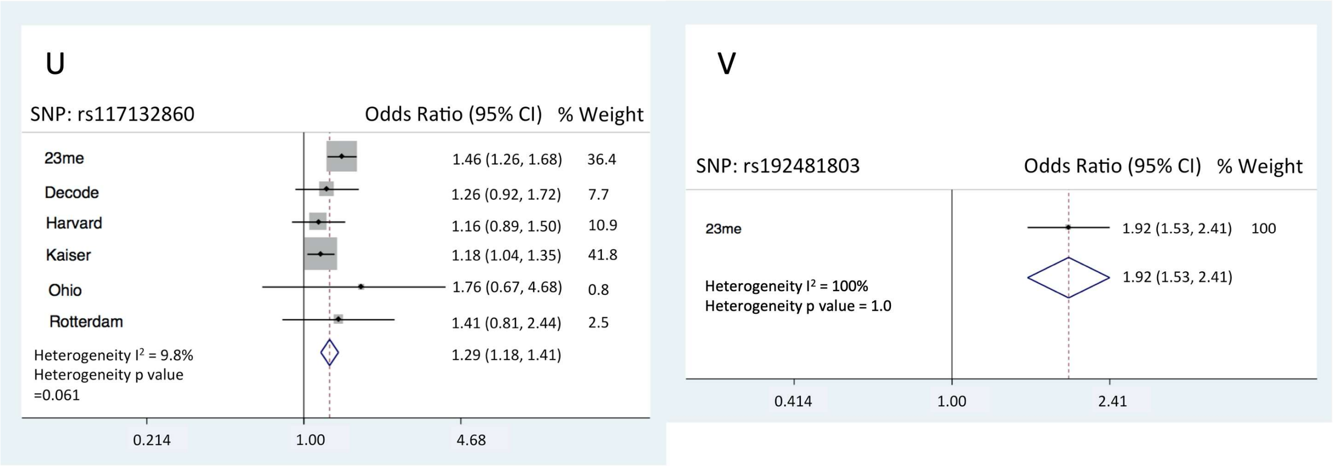

Supplementary Figure 3A-V: Forest plots of odds ratios for each SNP associated with SCC by individual study in the meta-analysis. The error bars represent the 95% confidence interval for the odds ratios for each study.

Supplementary Figure 4A-V:Regional association plots for novel SNP hits (Locus Zoom)

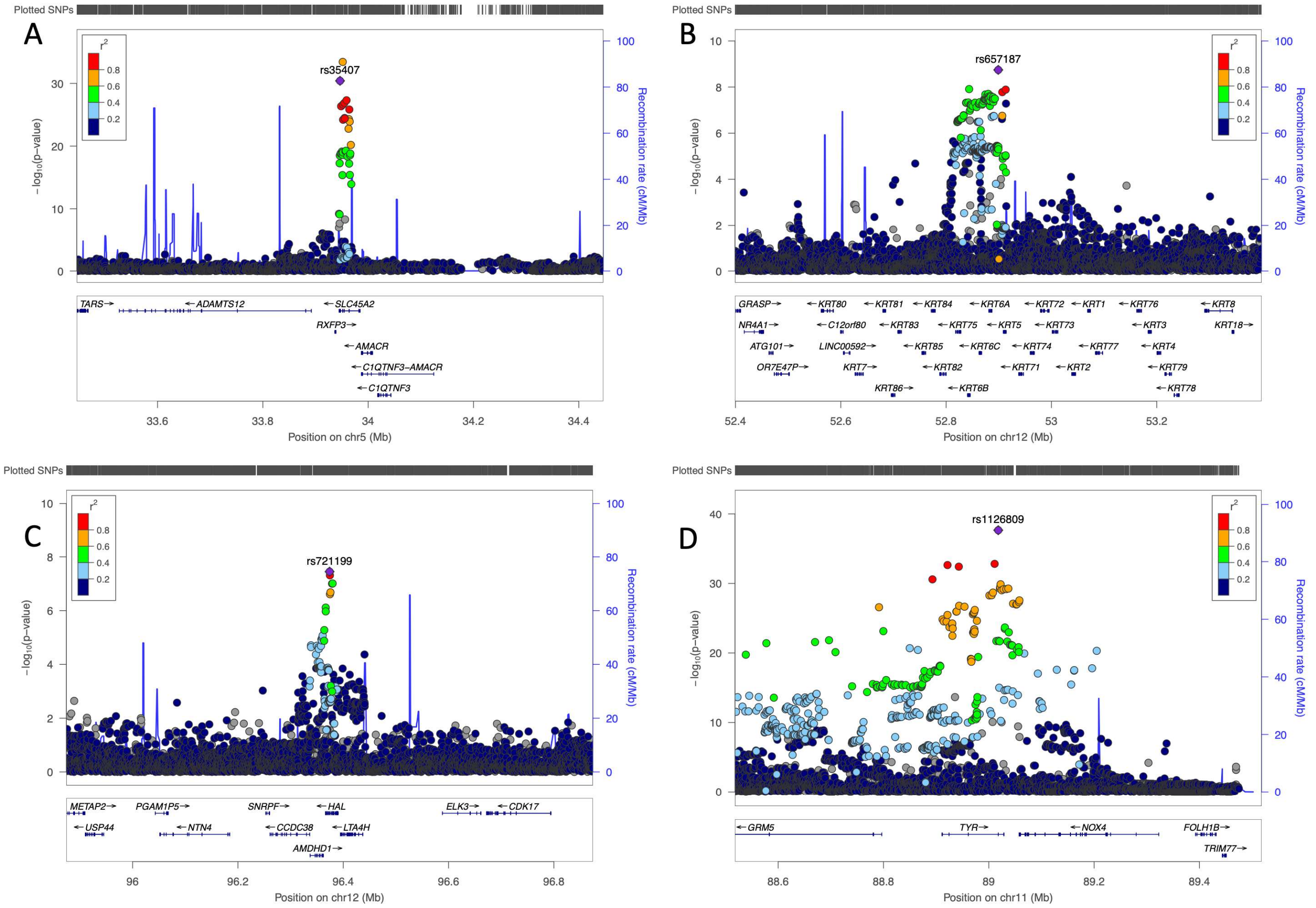

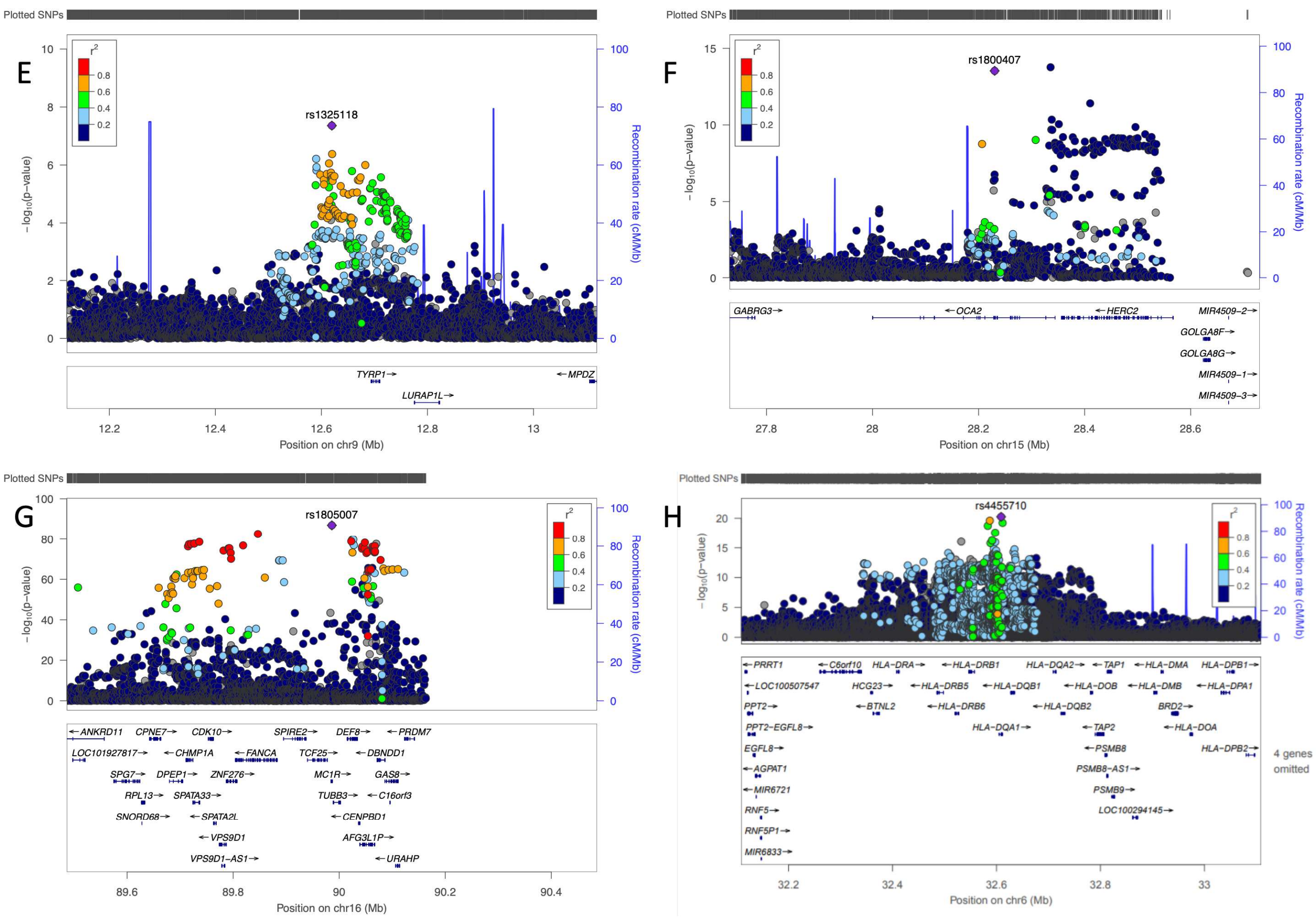

E
Plotted SNPs
rs1325118
$-\log_{10}$(p-value)
Recombination rate (cM/Mb)
TYRP1→
←MPDZ
LURAP1L→
Position on chr9 (Mb)
F
Plotted SNPs
rs1800407
GABRG3→
←OCA2
←HERC2
MIR4509-2→
GOLGA8F→
GOLGA8G→
MIR4509-1→
MIR4509-3→
Position on chr15 (Mb)
G
Plotted SNPs
rs1805007
←ANKRD11
CPNE7→
CDK10→
SPIRE2→
DEF8→
←PRDM7
LOC101927817→
←CHMP1A
←FANCA
TCF25→
←DBNDD1
SPG7→
DPEP1→
ZNF276→
MC1R→
GAS8→
RPL13→
SPATA33→
TUBB3→
←C16orf3
SNORD68→
←SPATA2L
←CENPBD1
←VPS9D1
AFG3L1P→
VPS9D1-AS1→
←URAHP
Position on chr16 (Mb)
H
Plotted SNPs
rs4455710
←PRRT1
←C6orf10
HLA-DRA→
←HLA-DRB1
HLA-DQA2→
←TAP1
←HLA-DMA
HLA-DPB1→
←LOC100507547
HCG23→
←HLA-DRB5
←HLA-DQB1
←HLA-DOB
←HLA-DMB
←HLA-DPA1
PPT2→
←BTNL2
←HLA-DRB6
←HLA-DQB2
BRD2→
PPT2-EGFL8→
HLA-DQA1→
←TAP2
←HLA-DOA
EGFL8→
←PSMB8
HLA-DPB2→
←AGPAT1
PSMB8-AS1→
←MIR6721
PSMB9→
RNF5→
LOC100294145→
RNF5P1→
MIR6833→
4 genes omitted
Position on chr6 (Mb)

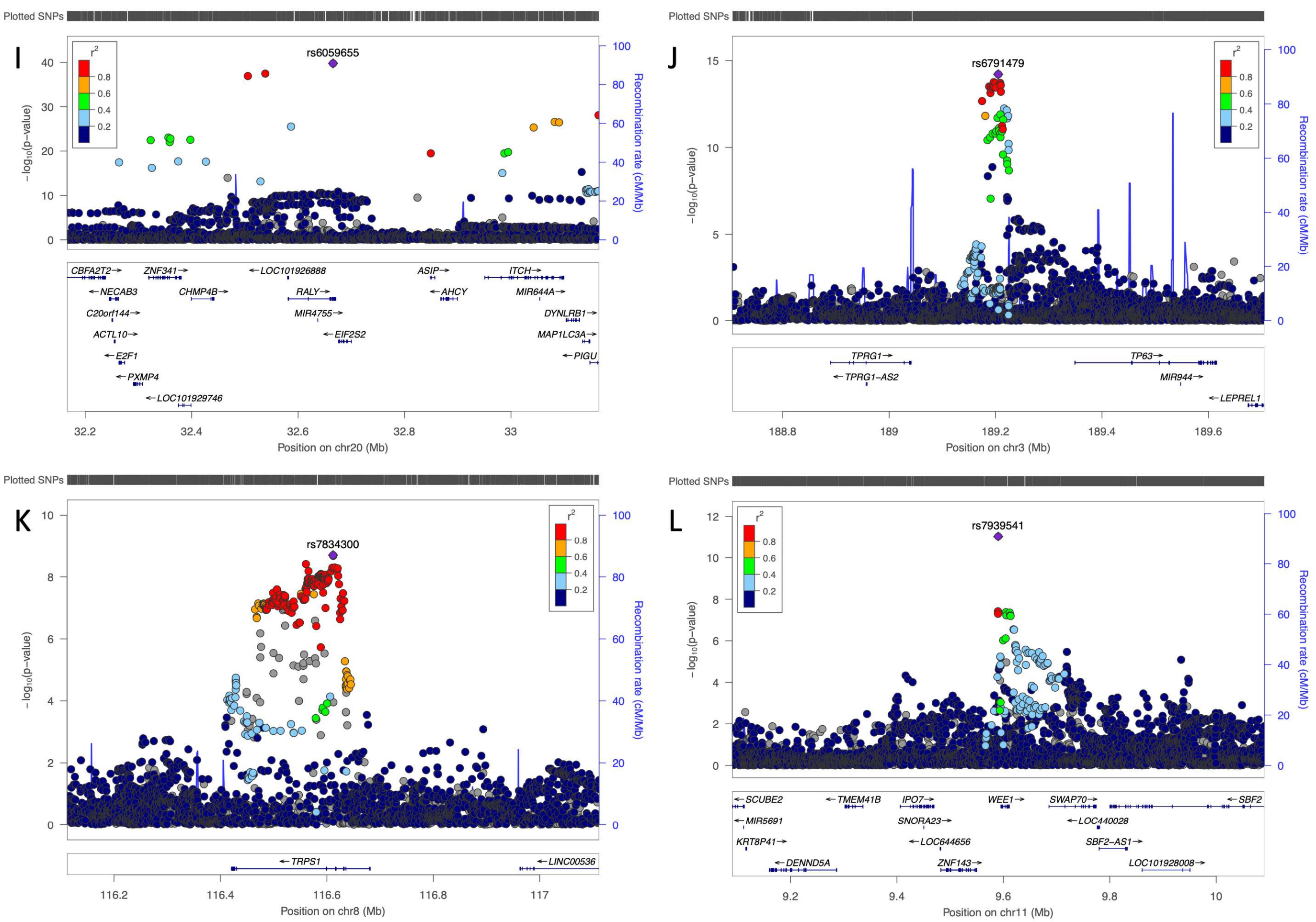

I
rs6059655
Position on chr20 (Mb)
J
rs6791479
Position on chr3 (Mb)
K
rs7834300
Position on chr8 (Mb)
L
rs7939541
Position on chr11 (Mb)
Plotted SNPs
−log10(p−value)
Recombination rate (cM/Mb)
CBFA2T2 ZNF341 LOC101926888 ASIP ITCH NECAB3 CHMP4B RALY AHCY MIR644A C20orf144 MIR4755 DYNLRB1 ACTL10 EIF2S2 MAP1LC3A E2F1 PIGU PXMP4 LOC101929746
TPRG1 TP63 TPRG1−AS2 MIR944 LEPREL1
TRPS1 LINC00536
SCUBE2 TMEM41B IPO7 WEE1 SWAP70 SBF2 MIR5691 SNORA23 LOC440028 KRT8P41 LOC644656 SBF2−AS1 DENND5A ZNF143 LOC101928008

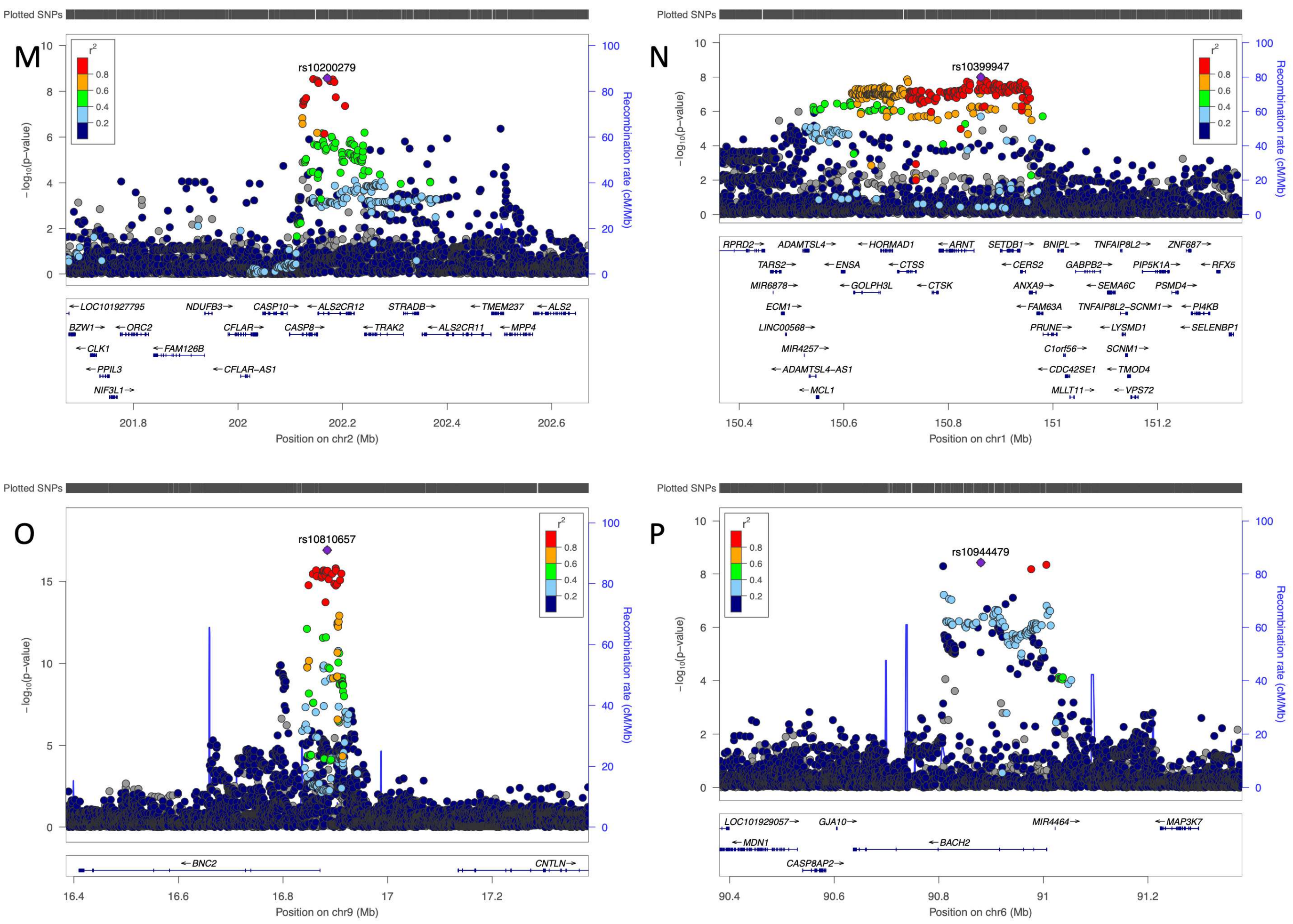

M
rs10200279
Plotted SNPs
−log10(p−value)
Recombination rate (cM/Mb)
r2
Position on chr2 (Mb)
LOC101927795
NDUFB3
CASP10
ALS2CR12
STRADB
TMEM237
ALS2
BZW1
ORC2
CFLAR
CASP8
TRAK2
ALS2CR11
MPP4
CLK1
FAM126B
PPIL3
CFLAR−AS1
NIF3L1
N
rs10399947
Position on chr1 (Mb)
RPRD2
ADAMTSL4
HORMAD1
ARNT
SETDB1
BNIPL
TNFAIP8L2
ZNF687
TARS2
ENSA
CTSS
CERS2
GABPB2
PIP5K1A
RFX5
MIR6878
GOLPH3L
CTSK
ANXA9
SEMA6C
PSMD4
ECM1
FAM63A
TNFAIP8L2−SCNM1
PI4KB
LINC00568
PRUNE
LYSMD1
SELENBP1
MIR4257
C1orf56
SCNM1
ADAMTSL4−AS1
CDC42SE1
TMOD4
MCL1
MLLT11
VPS72
O
rs10810657
Position on chr9 (Mb)
BNC2
CNTLN
P
rs10944479
Position on chr6 (Mb)
LOC101929057
GJA10
MIR4464
MAP3K7
MDN1
BACH2
CASP8AP2

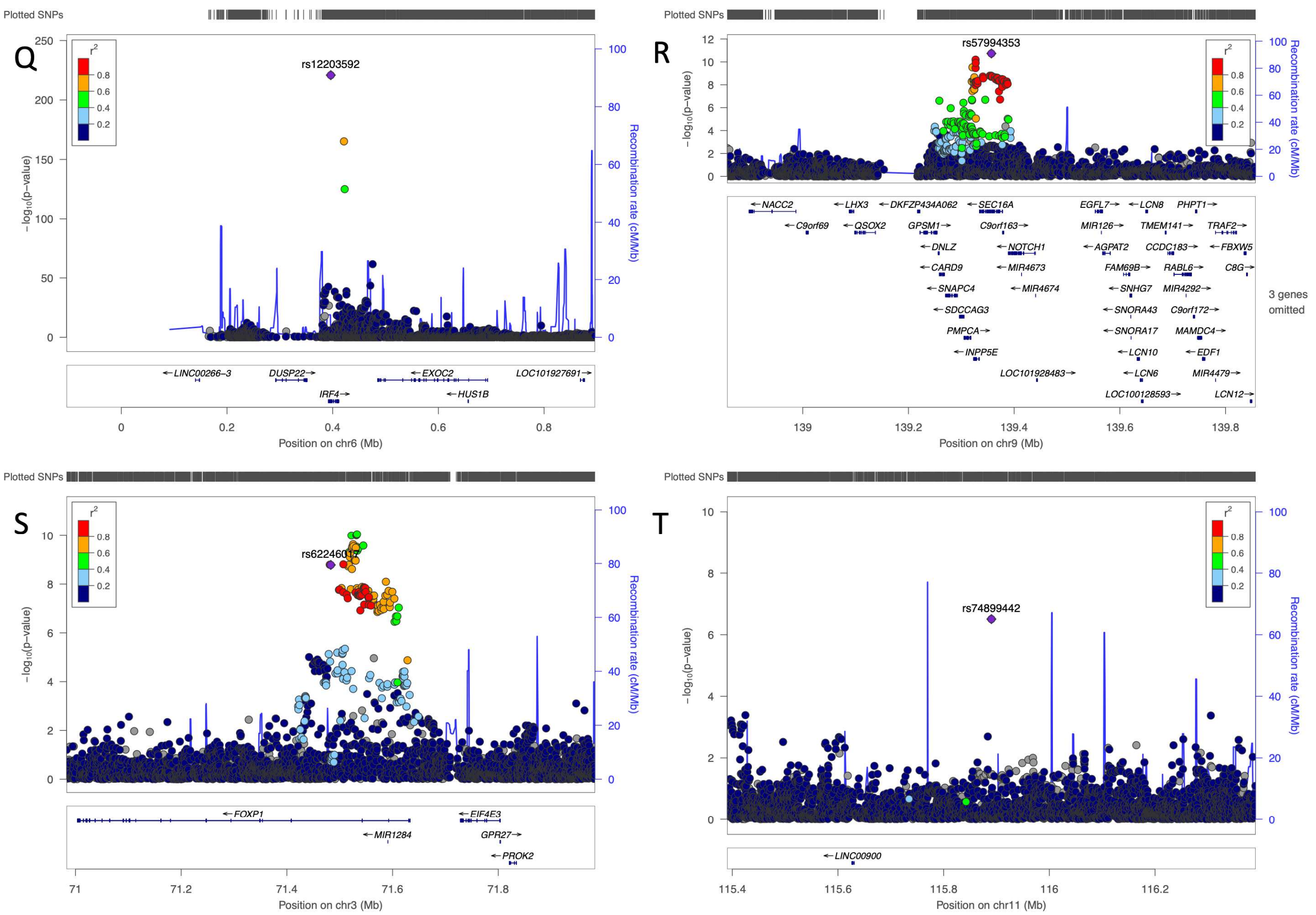
Q
R
S
T
Plotted SNPs
rs12203592
rs57994353
rs62246017
rs74899442
-log10(p-value)
Recombination rate (cM/Mb)
r2
Position on chr6 (Mb)
Position on chr9 (Mb)
Position on chr3 (Mb)
Position on chr11 (Mb)
LINC00266-3
DUSP22
IRF4
EXOC2
HUS1B
LOC101927691
NACC2
LHX3
DKFZP434A062
SEC16A
C9orf69
QSOX2
GPSM1
C9orf163
DNLZ
NOTCH1
CARD9
MIR4673
SNAPC4
MIR4674
SDCCAG3
PMPCA
INPP5E
LOC101928483
EGFL7
LCN8
PHPT1
MIR126
TMEM141
TRAF2
AGPAT2
CCDC183
FBXW5
FAM69B
RABL6
C8G
SNHG7
MIR4292
SNORA43
C9orf172
SNORA17
MAMDC4
LCN10
EDF1
LCN6
MIR4479
LOC100128593
LCN12
3 genes omitted
FOXP1
MIR1284
EIF4E3
GPR27
PROK2
LINC00900

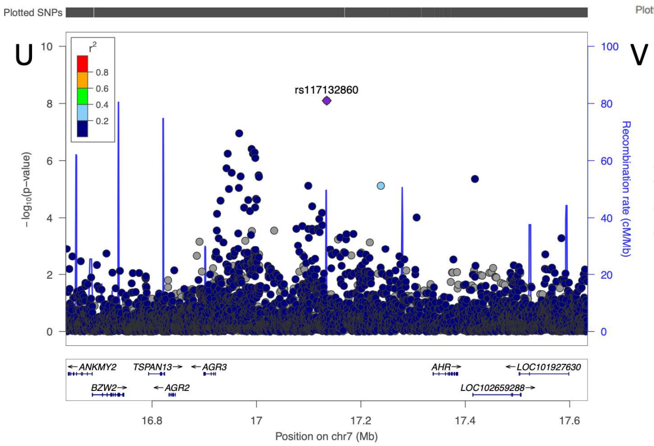

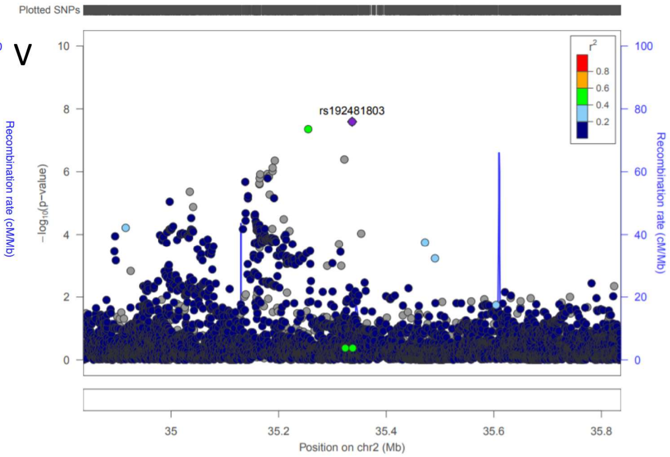

Supplementary Figures 4A-V: Regional association plots for novel SNP hits in GWAS meta-analysis.

Supplementary Table 1: Summary data for individual datasets by study.

Cohort	Cases	Controls	Genomic inflation (λ)	Number of SNPs	GWAS data Published:
**Rotterdam**	398	10,629	1.01	7,615,191	partially published (Siiskonen et al)
**23&Me**	6,579	280,558	1.05	11,468,464	published (Chahal et al)
**NHS/PHS**	2,287	30,966	1.03	8,316,372	partially published (Chahal et al)
**Kaiser**	7,701	60,166	1.05	8,723,724	partially published (Asgari et al)
**deCODE**	2,081	296,015	1.02	10,146,978	Unpublished
**Ohio**	103	1,715	1.01	9,289,320	Unpublished
**Total:**	19,149	680,049	1.06	13,607,369	

Supplementary Table 2: Demographic information for each cohort

Study	N	Male (%)	Age <30 yr	Age 30-45 yr	Age 46-60 yr	Age > 60 yr
**Rotterdam**						
**Cases**	398	192(48.2%)	0	0	69 (17.3%)	329 (82.7%)
**Controls**	10,629	4,451(41.9%)	0	0	3,770 (35.5%)	6,859(64.5%)
**23&Me**						
**Cases**	6,579	3510 (53.4%)	12 (0.2%)	199 (3.0%) 83,780	1,263 (19.2%)	5,105 (77.6%)
**Controls**	280,558	151,588 (54.0%)	39,838 (14.2%)	(29.9%)	76,833 (27.4%)	80,107 (28.6%)
**NHS/PHS**						
**Cases**	2,287	868 (37.9%)	10(0.4%)	918(40.0%)	1,098 (47.9%)	267 (11.6%)
**Controls**	30,966	8,687 (26.3%)	885 (2.7%)	17,602 (53.3%)	10,042 (30.4%)	2,491 (7.5%)
**Kaiser**						
**Cases**	7,701	4,420 (57.4%)	0 (0%)	45 (0.6%)	899 (11.7%)	6,757 (87.7%)
**Controls**	60,166	24,324 (40.4%)	918 (1.5%)	5,546 (9.2%)	21,090 (35.1%)	32,612 (54.2%)
**deCODE**						
**Cases**	2,081	935 (44.9%)	12 (0.6%)	74 (3.6%)	242 (11.6%)	1,753 (84.2%)
**Controls**	296,015	151,761(51.3%)	40,708 (13.8%)	64,323 (21.7%)	68,550 (23.2%)	122,434 (41.4%)
**Ohio**						
**Cases**	103	61 (59.2%)	0 (0%)	8 (7.7%)	28 (27.2%)	67 (65.0%)
**Controls**	1,715	499 (29.1%)	281 (16.4%)	516 (30.1%)	603 (35.2%)	315 (18.3%)
**Total:**						
**Cases**	19,149	9,986 (52.1%)	34 (0.2%)	1,244 (6.5%)	3,599 (18.8%)	14,278 (74.5%)
**Controls**	680,049	343,290 (50.3%)	82,630 (12.1%)	171,767 (25.2%)	180,888 (26.5%)	244,818 (35.9%)

Supplementary Table 3: Significant SNPs from previous cutaneous SCC GWAS.

SNP	Chr	Position	Cytoband	Gene	Major allele	Minor allele	MAF	Effect	Direction	p value	StdErr	Het I^2	Het Chi^2	Het	Het p value
rs192481803	2	35336564	2p22.3	[]	C	T	0.007	0.65	+, N, N, N, N, N	2.32E-07	0.12	NA	NA	NA	NA
rs62246017	3	71483084	3p13	FOXP1---[]---EIF4E3	G	A	0.325	0.07	+, +, -, +, -, +	1.65E-09	0.01	60.3%	12.58	5	0.03
rs6791479	3	189205032	3q28	TPRG1---[]---TP63	A	T	0.427	0.09	+, +, +, +, -, +	6.09E-15	0.01	71.4%	17.47	5	0.0037
rs35407	5	33946571	5p13.2	[SLC45A2]	G	A	0.042	-0.47	-, -, -, -, N, -	5.81E-31	0.04	83.5%	24.23	4	0.000072
rs4455710	6	32608858	6p21.32	[HLA-DQA1]	C	T	0.368	0.14	N, +, +, +, +, -	5.57E-21	0.02	45.8%	7.38	4	0.12
rs12203592	6	396321	6p25.3	[IRF4]	C	T	0.166	0.44	+, +, +, +, +, +	1.33E-221	0.01	79.1%	23.90	5	0.00023
rs117132860	7	17134708	7p21.1	AGR3---[]---AHR	G	A	0.021	0.25	+, +, +, +, +, +	7.97E-09	0.04	9.8%	5.54	5	0.35
rs10810657	9	16884586	9p22.2	BNC2--[]---CNTLN	A	T	0.404	-0.10	-, -, -, -, -, -	1.26E-17	0.01	0%	0.16	5	1.00
rs57994353	9	139356987	9q34.3	[SEC16A]	T	C	0.284	0.09	+, +, +, +, +, -	1.84E-11	0.01	52.5%	10.54	5	0.06
rs1126809	11	89017961	11q14.3	[TYR]	G	A	0.279	0.15	+, +, +, +, +, +	2.21E-38	0.01	0%	4.63	5	0.46
rs74899442*	11	115890279	11q23.3	CADM1---[]---BUD13	T	C	0.004	0.75	+, N, N, N, N, +, N	1.52E-07	0.13	NA	NA	NA	NA
rs1800407	15	28230318	15q13.1	[OCA2]	C	T	0.070	0.16	+, +, +, +, N, -	5.12E-14	0.02	54.9%	8.88	4	0.06
rs1805007	16	89986117	16q24.3	TCF25-[]-TUBB3	C	T	0.078	0.38	+, +, +, +, N, +	5.55E-87	0.02	4.0%	4.17	4	0.38
rs6059655	20	32665748	20q11.22	[RALY]	G	A	0.077	0.25	+, +, +, +, N, +	8.42E-40	0.02	50.3%	8.04	4	0.09

[o] degrees of freedom The minor allele is effect allele. MAF represents the minor allele frequency in the pooled meta-analysis.*rs74899442 MAF only met the MAF cutoff in the original dataset in which it was discovered; it did not meet the cutoff in the other cohorts used in this meta-analysis. However, we report these results in our supplementary to further support the significant association. Direction is listed in order for 23me, deCODE, NHS/HPFS, Kaiser, Ohio and Rotterdam. N means not included in analysis.

Supplementary Table 4: Novel SNPs reaching genome-wide significance in the SCC meta-analysis

SNP	Chr	Position	Locus	Gene	Major allele	Minor allele	MAF	Effect	Direction	p value	StdErr	Het I ^{2}	Het Chi squared	Het º	Het p value
rs10399947	1	150861960	1q21.3	ARNT--[]--SETDB1	G	A	0.36	-0.07	-, -, -, -, -, +	6.65E-09	0.01	0%	4.26	5	0.51
rs10200279	2	202170655	2q33.1	[ALS2CR12]	C	T	0.287	0.07	+, +, +, +, +, +	2.67E-09	0.01	0%	3.79	5	0.58
rs10944479	6	90880393	6q15	[BACH2]	G	A	0.189	-0.09	-, -, -, -, -, N	3.75E-09	0.02	0%	2.20	4	0.70
rs7834300	8	116611632	8q23.3	[TRPS1]	C	G	0.438	0.07	+, +, +, +, -, +	2.01E-09	0.01	0%	3.21	5	0.67
rs1325118	9	12619616	9p23	[]--TYRP1	T	C	0.304	-0.07	-, -, -, -, +, -	4.38E-08	0.01	0%	1.15	5	0.95
rs7939541	11	9590389	11p15.4	ZNF143--[]-WEE1	T	C	0.410	0.08	+, +, +, +, +, +	9.23E-12	0.01	2.5%	5.13	5	0.40
rs657187	12	52898985	12q13.13	KRT6A--[]-KRT5	A	G	0.420	-0.07	-, -, -, -, -, -	1.80E-09	0.01	0.0%	2.72	5	0.74
rs721199	12	96374057	12q23.1	[HAL]	C	T	0.463	-0.06	-, -, -, -, -, -	3.55E-08	0.01	58.9%	12.16	5	0.03

º degrees of freedom. Minor allele is effect allele. Minor allele frequency (MAF) is based on the pooled meta-analysis. Direction is listed in order for 23me, deCODE, NHS/HPFS, Kaiser, Ohio and Rotterdam. N means not included in analysis.

Supplementary Table 5: Per study results for the significant SNPS in SCC GWAS meta-analysis

SNP	23&Me			deCODE			NHS/PHS			Kaiser			Ohio			Rotterdam		
	Effect	Std Err	p val	Effect	Std Err	p val	Effect	Std Err	p val	Effect	Std Err	p val	Effect	Std Err	p val	Effect	Std Err	p val
rs10399947	-0.04	0.02	2.88E-02	-0.09	0.04	1.83E-02	-0.08	0.04	2.31E -02	-0.08	0.02	2.64E -06	-0.08	0.15	6.1E-01	0.01	0.08	9.36E-01
rs10200279	0.09	0.02	4.69E-06	0.10	0.04	1.04E-02	0.03	0.04	3.34E -01	0.05	0.02	4.77E -03	0.05	0.16	7.70E-01	0.12	0.08	1.17E-01
rs10944479	-0.11	0.03	1.84E-05	-0.10	0.04	2.35E-02	-0.10	0.05	5.99E -02	-0.07	0.02	2.71E -03	-0.25	0.22	2.50E-01	-	-	-
rs7834300	0.09	0.02	2.92E-06	0.08	0.04	2.59E-02	0.05	0.03	1.58E -01	0.05	0.02	3.78E -03	-0.05	0.15	7.45E-01	0.10	0.07	1.82E-01
rs1325118	-0.07	0.02	1.95E-04	-0.04	0.04	2.65E-01	-0.06	0.04	8.60E -02	-0.06	0.02	9.53E -04	0.01	0.16	9.69E-01	-0.12	0.08	1.34E-01
rs7939541	0.08	0.02	4.23E-06	0.11	0.04	1.75E-03	0.06	0.03	8.55E -02	0.06	0.02	3.73E -04	0.32	0.15	2.72E-02	0.03	0.07	7.04E-01
rs657187	-0.07	0.02	7.21E-05	-0.05	0.04	1.57E-01	-0.03	0.03	3.25E -01	-0.07	0.02	6.47E -05	-0.21	0.15	1.58E-01	-0.11	0.07	1.28E-01
rs721199	-0.04	0.02	2.77E-02	-0.03	0.03	3.42E-01	-0.11	0.03	1.08E -03	-0.07	0.02	1.23E -04	-0.49	0.15	9.21E-04	-0.07	0.07	3.65E-01

Supplementary Table 6: Fine mapping of significant loci in SCC GWAS meta-analysis

locus	cytoband	$N_{signals}$	SNP	pos	MAF	p	P_{joint}	C_{99}	C_{80}
1	1q21.3	1	rs10399947	150,861,960	0.368	1e-08	9.3e-09	273	158
2	2q33.1	1	rs10200279	202,170,655	0.287	2.7e-09	2.4e-09	20	9
3	2p22.3	0	(rs192481803)	(35,336,564)	(0.006)	(2.6e-08)			
4	3p13	1	rs62247166	71,532,738	0.479	8.9e-11	8.4e-11	66	17
5	3q28	1	rs9290890	189,196,565	0.427	1.7e-14	1.4e-14	17	9
6	5p13.2	1	rs16891982	33,951,693	0.055	3.7e-34	6.1e-34	1	1
8	6p25.3	2	rs12203592	396,321	0.166	1.3e-221	1.8e-192	1	1
			rs6935510	391,623	0.386	2.8e-43	8.5e-15	1	1
9	6q15	1	rs10944479	90,880,393	0.189	3.8e-09	3.6e-09	82	4
10	7p21.1	1	rs117132860	17,134,708	0.021	8e-09	8.3e-09	12	1
11	8q23.3	1	rs2737206	116,611,902	0.438	2.1e-09	2.3e-09	142	64
12	9p23	1	rs1325118	12,619,616	0.304	4.4e-08	4.9e-08	1990	16
13	9p22.2	2	rs62541919	16,863,364	0.414	2.1e-16	5.1e-16	21	13
			rs10962599	16,795,286	0.251	1.3e-10	3.3e-10	20	12
14	9q34.3	3	rs57994353	139,356,987	0.284	1.8e-11	1.7e-11	37	3
15	11q14.3	1	rs1126809	89,017,961	0.278	2.2e-38	2.8e-38	1	1
16	11q23.3	0	(rs74899442)	(115,890,279)	(0.004)	(1.5e-07)			
17	11p15.4	1	rs7939541	9,590,389	0.41	9.2e-12	8.6e-12	1	1
18	12q13.13	1	rs657187	52,898,985	0.42	1.8e-09	1.9e-09	113	31
19	12q23.1	1	rs721199	96,374,057	0.463	3.6e-08	3.1e-08	59	4
20	15q13.1	2	rs4778138	28,335,820	0.143	1.7e-14	1.9e-13	1	1
			rs1800407	28,230,318	0.070	2.9e-14	3.5e-13	1	1
22	20q11.22	1	rs6059655	32,665,748	0.076	1.7e-40	2.1e-40	1	1

$N_{signals}$: the number of distinct association signals in locus. (i) two loci were excluded: 6p21.32 as it was an HLA region and 16q24.3 as it is the MC1R locus; (ii) two loci have 0 signals as they were excluded from analysis due to being rare variants (iii) most loci have a single signal, as summarized in Table 1 and Supplementary Table 3. **SNP**: index SNP of the association signal from the conditional analysis. **pos**: index SNP position in bp. **MAF**: index SNP minor allele frequency. **P**: index SNP marginal P-value from GWAS meta-analysis. **P_{joint}**: index SNP P-value in the conditional joint analysis when all signals are included in the model. **C_{99}**: the size of 99% credible set. **C_{80}**: the size of 80% credible set. (*): For the two loci 3 and 16 the conditional analysis revealed no signals. The information of the lead SNP from GWAS meta-analysis for these loci is shown in brackets.(**)

Supplementary Table 7: Imputation quality by study for rs117132860

Study	Imputation R^2	Minor allele frequency	p value	Odds Ratio
**23me**	0.52	0.02	7.19E-07	1.46
**Decode**	0.99	0.01	1.48E-01	1.26
**Harvard**	0.65	0.02	2.72E-01	1.16
**Kaiser**	0.70	0.02	1.31E-02	1.18
**Ohio**	0.74	0.02	2.84E-01	1.76
**Rotterdam**	0.69	0.02	2.43E-01	1.41

Supplementary Table 8: Effect of each SNP on SCC on pigmentation traits

Rsid	Chr:Pos (B38)	Minor allele	Major allele	Risk allele	Gene	Variant Effect	Close Genes	Known Associations	p value	Effect	Phenotype
**rs1126809**	chr11: 89284793	A	G	G	TYR	Missense	TYR<br>NOX4<br>GRM5<br>FOLH1B<br>TRIM77<br>TRIM49	BCC[1]<br>SCC[2,3]<br>Sunburns[4]<br>Tanning[4]	1.80E-28	1.389	Sun sensitivity
									2.31E-28	1.565	Eye color (blue vs green)
									2.75E-06	1.252	Hair color (blond vs brown)
									5.19E-04	1.198	Eye color (blue vs brown)
**rs1800407**	chr15: 27985172	T	C	C	OCA2	Missense	OCA2<br>HERC2<br>LOC645202<br>GOLGA8F<br>MIR4509-2<br>GABRG3	SCC[2]	1.39E-71	0.197	Eye color (blue vs brown)
									1.11E-39	0.351	Eye Color (blue vs green)
									5.73E-10	0.522	Hair color (blond vs brown)
									0.00131	0.807	Freckles
**rs1805007**	chr16: 89919709	T	C	C	MC1R	Missense	MC1R<br>TUBB3<br>TCF25<br>DEF8<br>SPIRE2<br>CENPBD1	BCC [1,5]<br>SCC[2]<br>Hair color (Blond vs Brown) [6,7]<br>Freckles [6]<br>Hair color [4]<br>Hair color (Red vs not Red) [6,7]<br>Non-melanoma skin cancer[4]<br>Sun sensitivity[6]<br>Sunburns[4]<br>Tanning [4]	8.61E-202	7.726	Hair color (Red vs non-red)
									1.83E-121	3.51	Freckles
									3.45E-91	2.578	Sun sensitivity
									3.94E-16	1.958	Hair (blond vs brown)
									6.68E-05	0.789	Eye color (blue vs green)
**rs6059655**	chr20: 34077942	A	G	G	RALY	Intronic	RALY<br>EIF2S2<br>MIR4755<br>RALY-AS1<br>ASIP<br>AHCY	BCC[1]<br>SCC[2,3]<br>Facial pigmentation[8]<br>Skin color saturation[9]<br>Sun sensitivity[9]	3.67E-51	2.052	Sun sensitivity
									8.16E-47	2.127	Freckles
									4.27E-19	1.98	Hair color (red vs non-red)
									1.01E-12	1.787	Hair (blond vs brown)
**rs35407**	chr5: 33946466	A	G	G	SLC45A2	3' UTR	SLC45A2<br>RXFP3<br>AMACR<br>C1QTNF3-AMACR	BCC[1]<br>SCC[2]	3.87E-13	0.239	Hair (blond vs brown)
									2.29E-09	0.469	Sun sensitivity

							ADAMTS12<br>C1QTNF3		3.27E-09	0.448	Eye Color (blue vs green)
									0.000983	0.544	Eye color (blue vs brown)
**rs12203592**	chr6: 396321	T	C	C	IRF4	Intronic	IRF4<br>LOC105375104<br>DUSP22<br>EXOC2<br>LOC285766<br>LOC105374874	Actinic Keratosis[10]<br>BCC[1]<br>SCC[2,3]<br>Non-melanoma skin cancer[4]<br>Hair color (black vs blond, black vs red)[11]<br>Eye color[12]<br>Facial pigmentation[8]<br>Freckling[12]<br>Hair color_(not red)[7]<br>Hair_color [4,7,12,13]<br>Hair (greying) [13]<br>Skin color saturation[9]<br>Tanning [4]	1.26E-149	3.465	Freckles
									2.99E-103	0.177	Hair (blond vs brown)
									9.69E-55	1.91	Sun sensitivity
									1.05E-10	1.699	Eye color (blue vs brown)
**rs7834300**	chr8: 115599405	G	C	G	TRPS1	Intronic	TRPS1<br>LINC00536	None	0.000542	1.101	Sun Sensitivity
**rs1325118**	chr9: 12619616	C	T	T	.	Intergenic	TYRP1<br>LURAP1L-AS1<br>LURAP1L,MPDZ<br>LINC01235	None	8.97E-12	0.758	Eye Color (blue vs green)
									4.55E-05	0.802	Eye color (blue vs brown)
**rs10810657**	chr9: 16884588	T	A	T	.	Intergenic	BNC2<br>CNTLN<br>C9orf92<br>SH3GL2<br>CCDC171	BCC[1]<br>SCC[2]	6.27E-17	0.783	Freckles
									1.18E-11	0.826	Sun sensitivity
									9.49E-06	0.819	Hair (blond vs brown)

Supplementary Table 9: Effect of each SNP on SCC on high vs low photo-distributed sites

SNP	Major/ Minor Allele	High-Photo OR [CI]	p value	Low-Photo OR[CI]	p value	T-Test	T-test p value
**rs10200279**	C/T	1.10 [1.04-1.16]	2.33E-02	1.03 [0.94-1.12]	5.20E-01	1.19	0.23
**rs10399947**	G/A	0.94 [0.89-0.99]	2.15E-02	0.94 [0.86-1.02]	1.40E-01	0.05	0.96
**rs10810657**	A/T	0.91 [0.86-0.96]	4.43E-04	0.93 [0.85-1.01]	6.80E-02	-0.31	0.76
**rs10944479**	G/A	0.88 [0.85-0.95]	1.07E-03	0.98 [0.87-1.10]	730E-01	-1.01	0.31
**rs1126809**	G/A	1.14 [1.08-1.21]	7.32E-06	1.09 [0.99-1.19]	6.65E-01	0.87	0.38
**rs117132860**	G/A	1.28 [1.03-1.60]	2.77E-02	1.10 [0.78-1.15]	6.02E-01	0.84	0.40
**rs12203592**	C/T	1.39 [1.30-1.51]	5.25E-18	1.33 [1.18-1.50]	3.57E-06	0.80	0.42
**rs1325118**	T/C	0.96 [091-1.02]	1.71E-01	0.89 [0.82-0.98]	1.41E-02	1.30	0.19
**rs1800407**	C/T	1.17 [1.04-1.32]	7.30E-03	1.20 [1.01-1.44]	4.30E-02	-0.34	0.73
**rs1805007**	C/T	1.42 [1.30-1.55]	1.81E-14	1.34 [1.17-1.54]	3.99E-05	0.73	0.47
**rs35407**	G/A	0.90 [0.76-1.06]	2.06E-01	0.70 [0.53-0.92]	1.15E-02	1.56	0.12
**rs4455710**	C/T	1.11 [1.03-1.19]	6.56E-03	1.13 [1.00-1.26]	4.37E-02	-0.29	0.77
**rs57994353**	T/C	1.04 [0.98-1.10]	2.23E-01	1.03 [0.94-1.13]	4.86E-01	0.22	0.83
**rs6059655**	G/A	1.22 [1.12-1.34]	1.31E-05	1.29 [1.12-1.48]	3.35E-04	-0.52	0.60
**rs62246017**	G/A	1.02 [0.96-1.08]	5.62E-01	1.08 [0.99-1.18]	7.73E-02	-1.14	0.25
**rs657187**	A/G	0.96 [0.91-1.01]	9.13E-02	0.94 [0.87-1.03]	1.72E-01	0.26	0.79
**rs6791479**	A/T	1.11 [1.06-1.17]	5.80E-05	1.15 [1.06-1.25]	8.72E-04	-0.67	0.50
**rs721199**	C/T	0.96 [0.91-1.01]	8.24E-02	0.86 [0.79-0.93]	1.09E-04	2.22	0.03
**rs7834300**	C/G	1.07 [1.02-1.13]	6.53E-03	1.06 [0.97-1.15]	1.87E-01	0.21	0.83
**rs7939541**	T/C	1.11 [1.06-1.17]	3.33E-04	1.03 [0.95-1.12]	5.11E-01	1.46	0.14
**rs74899442**	T/C	0.99 [049-2.00]	9.79E-01	1.37 [0.43-4.32]	5.93E-01	-0.61	0.54

Minor allele is the effect allele.

Supplementary Table 10: Significant results (FDR < 0.10) of the LDSC regression analysis (baseline model) on 53 publicly available annotations.[1] Annotations with the 500bp windows have "500_0" at the end.
Columns: SNPs, the proportion of SNPs in Annotation; h2, the proportion of heritability explained by SNPs in Annotation (standard error); Enrich., the enrichment computed as ration between the two proportions (standard error); p, p-value for Enrichment computed by the jackknife resampling; p_{adj}, the adjusted p-value by the FDR method (q-value). Rows are ordered by p-values in the column, p.

**Annotation**	**SNPs**	**h^2**	**Enrich.**	**p**	**p_{adj}**
Coding_UCSC.extend.500_0	0.0646	0.432 (0.1286)	6.691 (1.992)	0.000848	0.032
SuperEnhancer_Hnisz.extend.500_0	0.1716	0.368 (0.0582)	2.147 (0.339)	0.001230	0.032
SuperEnhancer_Hnisz.500_0	0.1684	0.339 (0.1023)	2.010 (0.608)	0.010818	0.094
H3K4me1_Trynka.extend.500_0	0.6092	1.034 (0.1940)	1.697 (0.318)	0.005483	0.071
Repressed_Hoffman.extend.500_0	0.7191	0.390 (0.1149)	0.542 (0.160)	0.003498	0.061

1. Finucane, H.K. *et al.* Partitioning heritability by functional annotation using genome-wide association summary statistics. *Nat Genet* **47**, 1228-35 (2015).

Supplementary Table 11: Top 10 results of the LDSC regression analysis on 220 cell-type-specific publicly available annotations[2], conditioned on the baseline model (53 annotations).

Group	Annotation	SNPs	h^2	Enrich.	Z	p	p_{adj}
**Immune**	CD19_primary_(BI)	0.0409	0.355	8.68	2.33	0.0196	0.327
**Immune**	CD19_primary_(UW)	0.0410	0.359	8.75	2.48	0.0130	0.327
**Immune**	CD4+_CD25-_CD45RA+_naive_primary	0.0131	0.180	13.79	2.30	0.0214	0.327
**Immune**	CD8_memory_primary	0.0111	0.156	13.97	2.19	0.0288	0.337
**Immune**	CD19	0.0366	0.340	9.31	2.46	0.0138	0.327
**Immune**	CD4+_CD25+_CD127-_Treg_primary	0.0142	0.195	13.76	2.35	0.0186	0.327
**Immune**	CD4_naive_primary	0.0127	0.163	12.87	2.33	0.0198	0.327
**Immune**	CD3_primary	0.0319	0.190	5.96	1.58	0.1152	0.531
**Other**	Penis_foreskin_melanocyte_primary	0.0179	0.408	22.81	2.28	0.0227	0.327
**Immune**	CD4+_CD25+_CD127-_Treg_primary	0.0286	0.309	10.80	2.35	0.0189	0.327

Columns: SNPs, the proportion of SNPs in Annotation; h2, the proportion of heritability explained by SNPs in Annotation; Enrich., the enrichment computed as ration between the two proportions; Z, Z-score computed for each annotation independently by incorporating into the baseline model; p, p-value derived based on Z-score; p_{adj}, the adjusted p-value by the FDR method (q-value). Rows are ordered by p-values computed for Enrichment (column not shown).

Supplementary Table 12: Index SNPs that are eQTLs in skin tissue

The alternate allele in the human genome reference is the eQTL effect allele.[1]

Variant	Gene	Tissue	p value	Normalized Effect Size	Effect allele/Ref allele
rs10399947	*HORMAD1*	Skin - Not Sun Exposed (Suprapubic)	1.3e-75	0.95	A/G
rs10399947	*GOLPH3L*	Skin - Not Sun Exposed (Suprapubic)	2.4e-45	0.55	A/G
rs10399947	*CTSS*	Skin - Not Sun Exposed (Suprapubic)	1.5e-16	0.25	A/G
rs10399947	*SETDB1*	Skin - Not Sun Exposed (Suprapubic)	2.9e-13	-0.22	A/G
rs10399947	*ANXA9*	Skin - Not Sun Exposed (Suprapubic)	1.7e-12	0.19	A/G
rs10399947	*ECM1*	Skin - Not Sun Exposed (Suprapubic)	9.1e-5	-0.11	A/G
rs10399947	*RP11-54A4.2*	Skin - Not Sun Exposed (Suprapubic)	1.5e-4	-0.18	A/G
rs10399947	*HORMAD1*	Skin - Sun Exposed (Lower leg)	6.0e-103	1.0	A/G
rs10399947	*GOLPH3L*	Skin - Sun Exposed (Lower leg)	2.1e-73	0.74	A/G
rs10399947	*CTSS*	Skin - Sun Exposed (Lower leg)	4.6e-24	0.26	A/G
rs10399947	*SETDB1*	Skin - Sun Exposed (Lower leg)	4.7e-10	-0.18	A/G
rs10399947	*ANXA9*	Skin - Sun Exposed (Lower leg)	4.2e-9	0.13	A/G
rs10399947	*CERS2*	Skin - Sun Exposed (Lower leg)	5.1e-7	0.086	A/G
rs10200279	*CASP8*	Skin - Not Sun Exposed (Suprapubic)	1.6e-36	0.56	C/T
rs10200279	*ALS2CR12*	Skin - Not Sun Exposed (Suprapubic)	1.8e-11	-0.38	C/T
rs10200279	*CASP10*	Skin - Not Sun Exposed (Suprapubic)	1.6e-6	-0.19	C/T
rs10200279	*CASP8*	Skin - Sun Exposed (Lower leg)	1.3e-45	0.57	C/T
rs10200279	*ALS2CR12*	Skin - Sun Exposed (Lower leg)	4.3e-14	-0.37	C/T
rs10200279	*PPIL3*	Skin- Sun Exposed (Lower leg)	2.9e-6	-0.22	C/T
rs7939541	*WEE1*	Skin - Not Sun Exposed (Suprapubic)	1.3e-27	0.36	T/C
rs7939541	*snoU13*	Skin - Not Sun Exposed (Suprapubic)	1.6e-11	0.24	T/C
rs7939541	*WEE1*	Skin - Sun Exposed (Lower leg)	3.5e-17	0.28	T/C
rs7939541	*snoU13*	Skin - Sun Exposed (Lower leg)	3.5e-7	0.20	T/C
rs721199	*HAL*	Skin - Not Sun Exposed (Suprapubic)	1.2e-67	-0.47	C/T
rs721199	*RP11-256L6.3*	Skin - Not Sun Exposed (Suprapubic)	1.5e-4	-0.46	C/T
rs721199	*HAL*	Skin - Sun Exposed (Lower leg)	4.1e-79	-0.49	C/T
rs721199	*RP11-256L6.3*	Skin - Sun Exposed (Lower leg)	4.4e-9	-0.22	C/T
rs657187	*KRT6C*	Skin - Not Sun Exposed (Suprapubic)	8.1e-5	0.14	G/A
rs657187	*KRT6C*	Skin - Sun Exposed (Lower leg)	3.6e-19	0.36	G/A

1. The Gtex Consortium, Genetic effects on gene expression across human tissues. *Nature* **550**, 204-213 (2017).

Supplementary Table 13: Characterization of SNPs meeting 0.05 PPA cutoff at locus 2q33.1

SNP	PPA	eQTL in skin tissue	Regulatory motifs altered	Disease associations
**rs10200279**	0.12	*ALS2CR12*<br>*CASP8*<br>*CASP10*<br>*PPIL3*	CACD_1<br>Myb_2<br>Pou2f2_disc2 RREB-1_1, RREB-1_2<br>Zfp281<br>Zfp740	
**rs10931936**	0.11	*ALS2CR12*<br>*CASP8*<br>*CASP10*<br>*PPIL3*	EWSR1-FLI1<br>PU.1_known2<br>SPIB<br>TFII-I<br>TFIIA	Esophageal cancer[14]<br>Breast cancer[15]
**rs1830298**	0.099	*ALS2CR12*<br>*CASP8*<br>*CASP10*<br>*PPIL3*	AIRE_1<br>Dobox4	Breast cancer[16]
**rs3769818**	0.094	*ALS2CR12*<br>*CASP8*<br>*CASP10*<br>*PPIL3*	Arid5a<br>Foxj2<br>Zfp410	_
**rs7582362**	0.094	*ALS2CR12*<br>*CASP8*<br>*CASP10*<br>*PPIL3*	HNF1<br>PLZF	_
**rs6719014**	0.089	*ALS2CR12*<br>*CASP8*<br>*CASP10*<br>*PPIL3*	_	_
**rs700635**	0.081	*ALS2CR12*<br>*CASP8*<br>*CASP10*<br>*PPIL3*	CCNT2	Prostate cancer[17]<br>Basal cell carcinoma[18]
**rs6714430**	0.073	*ALS2CR12*<br>*CASP8*<br>*CASP10*<br>*PPIL3*	_	_
**rs6743068**	0.073	*ALS2CR12*<br>*CASP8*<br>*CASP10*<br>*PPIL3*	Irf<br>Pax-5<br>RXRA	_
**rs9677180**	0.071	*ALS2CR12*<br>*CASP8*<br>*CASP10*<br>*PPIL3*	Pax-4	_

Supplementary Table 14: Mutually adjusted analysis for rs657187 and 11170164 at 12q13.13

SNP	**Chr**	**Pos**	**Maj/Min**	**M AF**	**Unadj Effect[Std Error]**	**Unadj *P* value**	**Adj Effect [Std Error]**	**Adj *P* value**
**rs657187**	12	52505201	A/G	0.42.	-0.07 [0.01]	1.803e-09	-0.053 [0.01]	2.28E-06
**rs11170164**	12	52519884	C/T	0.08	0.11 [0.02]	5.218e-08	0.082 [0.02]	5.67E-05

Minor Allele is Effect Allele. Chr = Chromosome, Pos= Position, Ref = Reference , Maj/Min = Major Allele/Minor Allele AF = Allele Frequency, Unadj = Unadjusted, Adj = Adjusted, Std = Standard

Supplementary References

1. Chahal, H.S. *et al.* Genome-wide association study identifies 14 novel risk alleles associated with basal cell carcinoma. *Nat Commun* **7**, 12510 (2016).
2. Chahal, H.S. *et al.* Genome-wide association study identifies novel susceptibility loci for cutaneous squamous cell carcinoma. *Nat Commun* **7**, 12048 (2016).
3. Asgari, M.M. *et al.* Identification of Susceptibility Loci for Cutaneous Squamous Cell Carcinoma. *J Invest Dermatol* **136**, 930-7 (2016).
4. Zhang, M. *et al.* Genome-wide association studies identify several new loci associated with pigmentation traits and skin cancer risk in European Americans. *Hum Mol Genet* **22**, 2948-59 (2013).
5. Nan, H. *et al.* Genome-wide association study identifies novel alleles associated with risk of cutaneous basal cell carcinoma and squamous cell carcinoma. *Hum Mol Genet* **20**, 3718-24 (2011).
6. Sulem, P. *et al.* Genetic determinants of hair, eye and skin pigmentation in Europeans. *Nat Genet* **39**, 1443-52 (2007).
7. Tung, J.Y. *et al.* Efficient replication of over 180 genetic associations with self-reported medical data. *PLoS One* **6**, e23473 (2011).
8. Jacobs, L.C. *et al.* A Genome-Wide Association Study Identifies the Skin Color Genes IRF4, MC1R, ASIP, and BNC2 Influencing Facial Pigmented Spots. *J Invest Dermatol* **135**, 1735-1742 (2015).
9. Liu, F. *et al.* Genetics of skin color variation in Europeans: genome-wide association studies with functional follow-up. *Hum Genet* **134**, 823-35 (2015).
10. Jacobs, L.C. *et al.* IRF4, MC1R and TYR genes are risk factors for actinic keratosis independent of skin color. *Hum Mol Genet* **24**, 3296-303 (2015).
11. Han, J. *et al.* A genome-wide association study identifies novel alleles associated with hair color and skin pigmentation. *PLoS Genet* **4**, e1000074 (2008).
12. Eriksson, N. *et al.* Web-based, participant-driven studies yield novel genetic associations for common traits. *PLoS Genet* **6**, e1000993 (2010).
13. Adhikari, K. *et al.* A genome-wide association scan in admixed Latin Americans identifies loci influencing facial and scalp hair features. *Nat Commun* **7**, 10815 (2016).
14. Zhao, X.K. *et al.* Shared susceptibility loci at 2q33 region for lung and esophageal cancers in high-incidence areas of esophageal cancer in northern China. *PLoS One* **12**, e0177504 (2017).
15. Sueta, A. *et al.* A genetic risk predictor for breast cancer using a combination of low-penetrance polymorphisms in a Japanese population. *Breast Cancer Res Treat* **132**, 711-21 (2012).
16. Lin, W.Y. *et al.* Identification and characterization of novel associations in the CASP8/ALS2CR12 region on chromosome 2 with breast cancer risk. *Hum Mol Genet* **24**, 285-98 (2015).
17. Stacey, S.N. *et al.* Insertion of an SVA-E retrotransposon into the CASP8 gene is associated with protection against prostate cancer. *Hum Mol Genet* **25**, 1008-18 (2016).
18. Stacey, S.N. *et al.* New basal cell carcinoma susceptibility loci. *Nat Commun* **6**, 6825 (2015).
