# Supplementary material for: Genome-wide meta-analysis identifies eight new susceptibility loci for cutaneous squamous cell carcinoma
Source: Nat Commun. 2020 Feb 10;11:820. doi: 10.1038/s41467-020-14594-5 (PMC7010741; doi:10.1038/s41467-020-14594-5)
Supplement: Supplementary file 3 — Reporting Summary [file 41467_2020_14594_MOESM3_ESM.pdf]

nature research

Corresponding author(s): Kavita Sarin, Jiali Han

Last updated by author(s): 1/14/2020

Reporting Summary

Nature Research wishes to improve the reproducibility of the work that we publish. This form provides structure for consistency and transparency in reporting. For further information on Nature Research policies, see Authors & Referees and the Editorial Policy Checklist.

Statistics

For all statistical analyses, confirm that the following items are present in the figure legend, table legend, main text, or Methods section.

n/a	Confirmed	
☐	☒	The exact sample size (*n*) for each experimental group/condition, given as a discrete number and unit of measurement
☒	☐	A statement on whether measurements were taken from distinct samples or whether the same sample was measured repeatedly
☐	☒	The statistical test(s) used AND whether they are one- or two-sided<br>*Only common tests should be described solely by name; describe more complex techniques in the Methods section.*
☐	☒	A description of all covariates tested
☐	☒	A description of any assumptions or corrections, such as tests of normality and adjustment for multiple comparisons
☐	☒	A full description of the statistical parameters including central tendency (e.g. means) or other basic estimates (e.g. regression coefficient) AND variation (e.g. standard deviation) or associated estimates of uncertainty (e.g. confidence intervals)
☒	☐	For null hypothesis testing, the test statistic (e.g. *F*, *t*, *r*) with confidence intervals, effect sizes, degrees of freedom and *P* value noted<br>*Give P values as exact values whenever suitable.*
☒	☐	For Bayesian analysis, information on the choice of priors and Markov chain Monte Carlo settings
☒	☐	For hierarchical and complex designs, identification of the appropriate level for tests and full reporting of outcomes
☒	☐	Estimates of effect sizes (e.g. Cohen's *d*, Pearson's *r*), indicating how they were calculated

Our web collection on statistics for biologists contains articles on many of the points above.

Software and code

Policy information about availability of computer code

Data collection	Raw RNA-seq data obtained from GSE84194
Data analysis	Fixed-effects meta-analysis was conducted using the METAL software. We identified the closest related gene and evidence of regulatory function using HaploReg v4.1

For manuscripts utilizing custom algorithms or software that are central to the research but not yet described in published literature, software must be made available to editors/reviewers. We strongly encourage code deposition in a community repository (e.g. GitHub). See the Nature Research guidelines for submitting code & software for further information.

Data

Policy information about availability of data

All manuscripts must include a data availability statement. This statement should provide the following information, where applicable:

- Accession codes, unique identifiers, or web links for publicly available datasets
- A list of figures that have associated raw data
- A description of any restrictions on data availability

Data from 23andMe, Inc were made available under a data use agreement that protects participant privacy. Please contact dataset-request@23andme.com or visit research.23andMe.com/collaborate for more information and to apply to access the data. Precomputed rankings and P-values for the top 10,000 SNPs included in the GWAS meta-analysis are available in the figshare repository https://doi.org/10.6084/m9.figshare.11588325. Any additional data (beyond those included in the main text and Supplementary Information) that support the findings of this study are available from the corresponding author upon request.

Field-specific reporting

Please select the one below that is the best fit for your research. If you are not sure, read the appropriate sections before making your selection.

☒ Life sciences ☐ Behavioural & social sciences ☐ Ecological, evolutionary & environmental sciences

For a reference copy of the document with all sections, see nature.com/documents/nr-reporting-summary-flat.pdf

Life sciences study design

All studies must disclose on these points even when the disclosure is negative.

Sample size	The GWAS meta-analysis is comprised of six international cohorts (Supplementary Table 1). The GWAS dataset from the personal genetic company 23andMe Inc. encompassed 6,579 SCC cases and 280,558 controls of European ancestry who consented to participate in research. The GWAS data set from the Nurse's Health Study (NHS)/ Health Professionals Follow-Up Study (HPFS) consisted of 2,287 SCC cases and 30,966 controls of European ancestry. The 23andMe data and some of the NHS/HPFS data were used in a previously reported GWAS.4 Kaiser Permanente Northern California contributed a GWAS set encompassing 7,701 cases with incident SCC and 60,166 controls of European ancestry. Some of these data were used in another previously reported GWAS.5,6 GWAS data from the deCODE study encompassed 2,081 SCC cases and 296,015 controls of European ancestry. The Rotterdam study contributed a GWAS data set consisting of 398 cases with SCC and 10,629 controls of European ancestry. The Ohio study included GWAS data on 103 SCC cases and 1,715 controls of European ancestry
Data exclusions	SNPs with imputation quality R2 < 0.3 in any dataset were excluded from that individual study prior to meta-analysis. For each study, SNPs with low expected minor allele counts in cases (overall minor allele frequency times number of cases < 10) were also removed before meta-analysis.
Replication	No validation cohort (one stage GWAS)
Randomization	N/A
Blinding	N/A

Reporting for specific materials, systems and methods

We require information from authors about some types of materials, experimental systems and methods used in many studies. Here, indicate whether each material, system or method listed is relevant to your study. If you are not sure if a list item applies to your research, read the appropriate section before selecting a response.

Materials & experimental systems

n/a	Involved in the study
☒	☐ Antibodies
☒	☐ Eukaryotic cell lines
☒	☐ Palaeontology
☒	☐ Animals and other organisms
☐	☒ Human research participants
☒	☐ Clinical data

Methods

n/a	Involved in the study
☒	☐ ChIP-seq
☒	☐ Flow cytometry
☒	☐ MRI-based neuroimaging

Human research participants

Policy information about studies involving human research participants

Population characteristics	The GWAS meta-analysis is comprised of six international cohorts (Supplementary Table 1). The GWAS dataset from the personal genetic company 23andMe Inc. encompassed 6,579 SCC cases and 280,558 controls of European ancestry who consented to participate in research. The GWAS data set from the Nurse's Health Study (NHS)/ Health Professionals Follow-Up Study (HPFS) consisted of 2,287 SCC cases and 30,966 controls of European ancestry. The 23andMe data and some of the NHS/HPFS data were used in a previously reported GWAS.4 Kaiser Permanente Northern California contributed a GWAS set encompassing 7,701 cases with incident SCC and 60,166 controls of European ancestry. Some of these data were used in another previously reported GWAS.5,6 GWAS data from the deCODE study encompassed 2,081 SCC cases and 296,015 controls of European ancestry. The Rotterdam study contributed a GWAS data set consisting of 398 cases with SCC and 10,629 controls of European ancestry. The Ohio study included GWAS data on 103 SCC cases and 1,715 controls of European ancestry.
Recruitment	Meta-analysis GWAS. Each site provide summary GWAS data.
Ethics oversight	This is a meta-analysis and summary GWAS data was provided for each cohort.

Note that full information on the approval of the study protocol must also be provided in the manuscript.
